# Structural Necessity of Indole C5-*O*-Substitution of *seco*-Duocarmycin Analogs for Their Cytotoxic Activity

**DOI:** 10.3390/molecules15117971

**Published:** 2010-11-08

**Authors:** Taeyoung Choi, Eunsook Ma

**Affiliations:** College of Pharmacy, Catholic University of Daegu, Hayang, 712-702, Korea

**Keywords:** duocarmycin, Indole C5-*O*-substituted s*eco*-CI, cytotoxicity, DNA minor groove alkylation

## Abstract

A series of *racemic* indole C5-*O-*substituted *seco*-cyclopropylindole (*seco*-CI) compounds **1**-**5** were prepared by coupling in the presence of EDCI of 1-(*tert*-butyloxycarbonyl)-3-(chloromethyl)indoline (seg-A) with 5-hydroxy-, 5-*O*-methylsulfonyl, 5-*O*-aminosulfonyl, 5-*O*-(*N,N*-dimethylaminosulfonyl)- and 5-*O*-benzyl-1*H*-indole-2-carboxylic acid as seg-B. Compounds **1-5** were tested for cytotoxic activity against four human cancer cell lines (COLO 205, SK-MEL-2, A549, and JEG-3) using a MTT assay. Compounds **2** and **3** with small sized sulfonyl substituents like 5-*O*-methylsulfonyl and 5-*O*-aminosulfonyl exhibit a similar level of activity as doxorubicin against all cell lines tested.

## 1. Introduction

**Figure 1:**
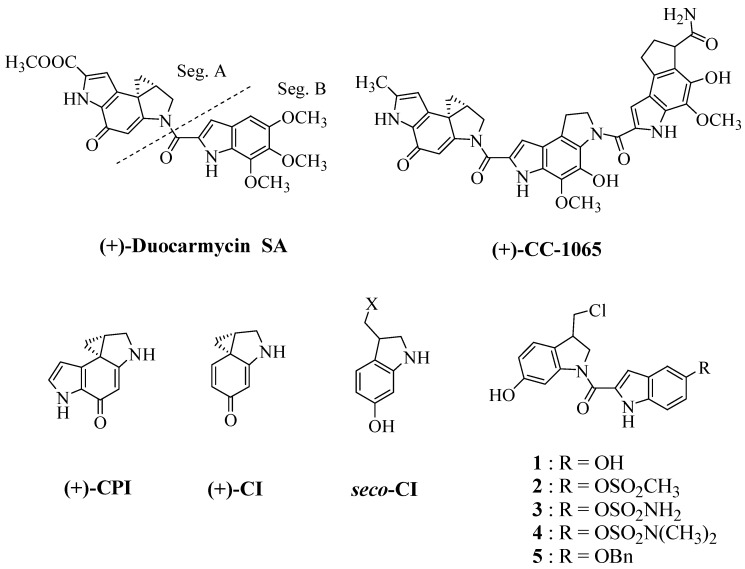
Structures of duocarmycin SA, CC-1065, and compounds **1**-**5**.

Duocarmycins (DUMs) A, B1, B2, C1, C2, D1, D2 and SA and CC-1065 are members of an extremely potent group of antitumor antibiotics isolated from *Streptomyces* sp. that contain the cyclopropa[*c*]pyrrolo[3,2-*e*]indole (CPI) moiety as a common pharmacophore [1,2,3]. DUM B1, B2, C1, C2, D1 and D2 are a *seco*-type of DUM A and are chemically more stable than DUM A (Figure 1). 

The cytotoxicity of DUMs and CC-1065 results from the reaction of the CPI moiety with adenine-N3 groups in the minor groove of AT-rich sequences of DNA [4]. Even though (+)-CC-1065 has potential cytotoxic properties, its use as an anticancer drug is hampered by delayed lethal toxicity to animals at therapeutic doses [5]. DUM SA seems to be superior to CC-1065 due to the lack of a delayed fetal hepatotoxicity like that with (+)-CC-1065 [6]. Moreover, DUM SA is the most hydrolytically stable member of this class of compounds [6,7,8], but it is toxic to the bone marrow [9]. 

**Scheme 1:**
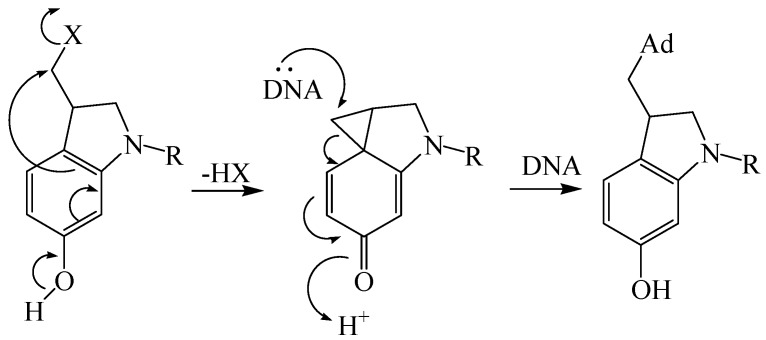
Proposed mechanism of activation and DNA alkylation of the *seco*-CI moiety [14].

Therefore, there is a strong interest in the design and development of novel analogs of duocarmycin that effectively kill cancer cells and have reduced toxicity to the host. One attempt [10,11] to design novel analogs involves the synthesis of a spirocyclic 1,2,7,7a-tetrahydrocycloprop[1,2-*c*]indol-4-one [(CI-numbering), CI] as the minimum size pharmacophore [12], which can be formed by ring closure of a *seco*-CI parent (Scheme 1). *Seco* compounds possess a similar alkylating selectivity and efficiency as the corresponding natural product (+)-enantiomers [13] since they can form the cyclopropane moiety *in situ* by an intramolecular Winstein cyclization [14]. 

Most duocarmycins exhibit more pronounced distinctions between enantiomers, but the spirocyclic 1,2,7,7a-tetrahydro-3-[(5,6,7-trimethoxyindol-2-yl)carbonyl]cycloprop[1,2-c]indol-4-one (CI-TMI) has been observed to show similar biological potency and relative DNA alkylation efficiency [13,15]. There is no difference of cytotoxic activity between (+)-CI and (-)-CI enantiomers, therefore no resolution is required to obtain a more active enantiomer.

Some previous results indicated that the seg-A ring structure influences the electrophilicity of cyclopropane [16]. On the other hand, the seg-B one has been considered to play some important roles in noncovalent binding to DNA [17,18,19]. Especially, the C5 position of seg-B was known as the most important site potentiating the cytotoxic activity. An adequate indole C5 substituent may not only extend the rigid length of the compound enhancing the DNA binding and thus accelerating the rate of DNA alkylation, but also benefit close contacts within a minor groove hydrophobic pocket [20]. Boger reported that a single 5-methoxy group on the indole as a minor groove binding unit was sufficient to maintain potency and a series of dimethylaminoethoxy group substituted analogs retained the cytotoxicity of the TMI analog, while providing increased aqueous solubility [16,21].

Indole C5 sulfone derivatives were synthesized and evaluated against the L1210 cell line and as a result, the C5 methanesulfonyl derivatives proved exceptionally potent, being 15–20 fold more active than the corresponding thiomethyl or methoxy derivatives [20]. Herein we describe the synthesis and cytotoxic activity of indole-C5-*O*-substituted and *O*-sulfonyl *seco*-CI analogs as different minor groove binding side chains.

## 2. Results and Discussion

### 2.1. Synthesis

**Scheme 2:**
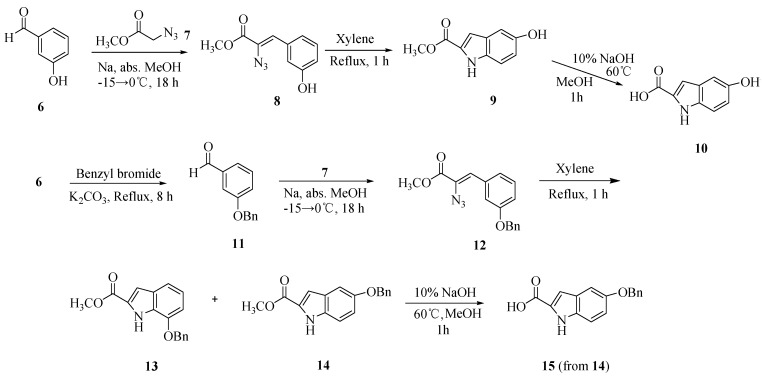
Synthesis of 5-hydroxy- and 5-*O*-benzyl-1*H*-indole-2-carboxylic acids **10** and **15**.

In order to obtain indole C5-*O*-substituted-*seco*-CI derivatives, we synthesized 5-hydroxy- (**10**) and 5-benzyloxy-1*H*-indole-2-carboxylic acid (**15**) from 3-hydroxybenzaldehyde (**6**) (Scheme 2). Methyl azidoacetate (**7**) was obtained from the reaction of methyl chloroacetate and sodium azide [22]. According to the method of Fukuda *et al.* [23], 3-hydroxybenzaldehyde was reacted with **7** to afford the azidocinnamate ester **8**. This compound was refluxed in xylene to synthesize methyl 5-hydroxy-1*H*-indole-**2**-carboxylic acid (**9**, 42%) which was hydrolyzed to afford 5-hydroxy-1*H*-indole-2-carboxylic acid (**10**). From compound **10**, we tried to introduce a benzyl group at the 5-hydroxy position, but obtained instead a *N-* and *O*-dibenzylated compound. In order to obtain the *O*-benzylated compound, treatment of **6** with benzyl bromide and potassium carbonate yielded **11**, which was subsequently converted to regioisomeric indoles **13** and **14** in 40% and 45% yield using the same conditions as for the preparation of **9**. Coowar *et al*. reported that cyclization of an unsymmetrical azidocinnamate ester provided two regioisomers in proportions depending upon the substitution position. They obtained the 5-methoxy- and the 7-methoxyindole carboxylates from the 3-methoxy-azidocinnamate in a 1:1 ratio, whereas the 5,6-dimethoxyindole carboxylate was obtained from the 3,4-dimethoxy derivative in a high yield [24]. Compounds **8** and **11** have substituents at same position, therefore the outcome of cyclization of these compounds seemed to be dependent upon the substituent. Alkaline hydrolysis of **14** gave 5-benzyloxy-1*H*-indole-carboxylic acid (**15**).

**Scheme 3:**
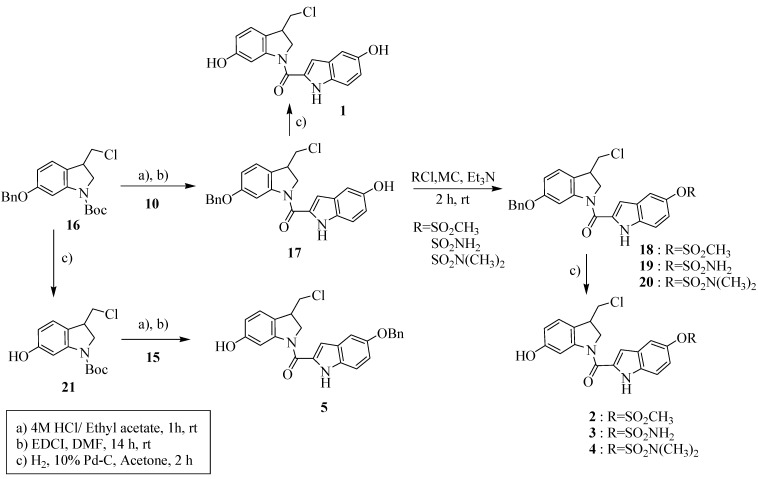
Synthesis of indole C5-*O*-substituted *seco*-CI derivatives **1-5**.

Scheme 3 shows a refinement of the synthesis that allows incorporation of various minor groove targeting units. We synthesized *seco-*5-benzyloxy-1-chloromethyl-*N-*BOC-indoline (*seco*-*N*-BOC-CI, **16**) from 4-amino-3-nitrophenol in six steps with an overall yield of 37% by the method reported in our earlier paper [25]. 

Deprotection of compound **16** under acidic conditions yielded the amine which was coupled with 5-hydroxyindole-2-carboxylic acid (**10**) in the presence of 1-ethyl-3-(3-dimethylaminopropyl) carbodiimide (EDCI) to give **17**. Debenzylation of **17** by catalytic hydrogenation in the presence of 10% Pd-C afforded compound **1** in 60% yield. Compound **17** was then reacted with methylsulfonyl-, aminosulfonyl-, or *N,N*-dimethylaminosulfonyl chloride afford **18**, **19**, and **20** in 92%, 62%, and 72% yield, respectively. Debenzylation of these compounds by catalytic hydrogenation in the presence of 10% Pd-C yielded compounds **2**, **3** and **4** in 50%, 53%, and 66% yield, respectively. In order to obtain indole-C5-*O*-benzyl-*seco*-CI, **16** was treated with 10% Pd-C to yield **21** and then reacted with 5-benzyloxy-1*H*-indole-2-carboxylic acid (**15**) in the presence of EDCI to give **5** in 83% yield.

### 2.2. Cytotoxic activity

**Figure 2:**
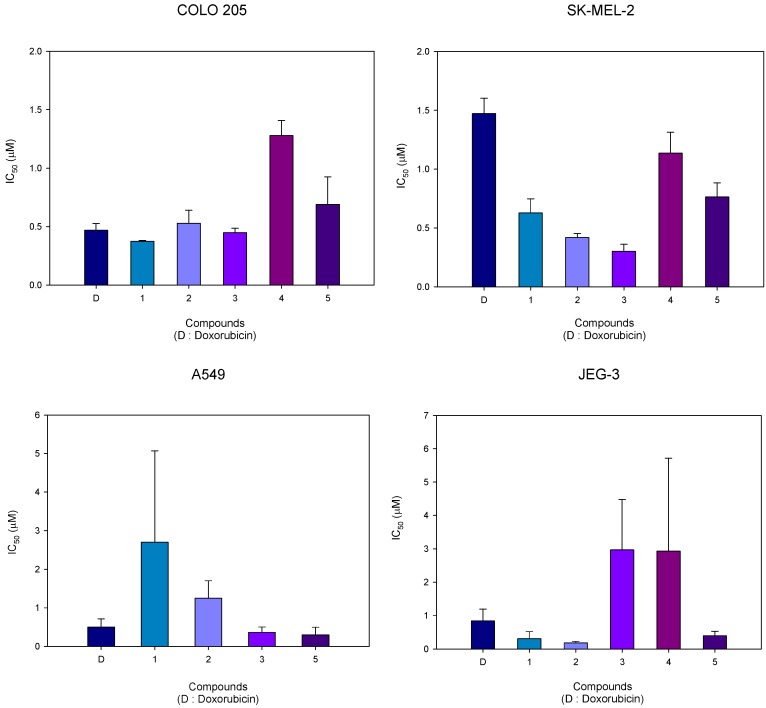
Cytotoxic activity of indole C5-*O*-substituted *seco*-CI derivatives **1-5**.

Cytotoxic activity was determined as IC_50_ values in four cell lines (COLO 205, SK MEL-2, A549 and JEG-3) for a 48 h drug exposure using a growth inhibition microassay. The results of the evaluation of the *in vitro* cytotoxic activity of compounds **1-5** are presented in Table 1 and Figure 2. 

**Table 1:** Cytotoxicity of indole C5-*O*-substituted *seco*-CI derivatives against cancer cell lines by MTT assay.

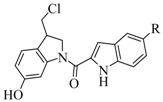					
Compound	R	IC_50_ (µM)^a)^			
		COLO 205	SK-MEL-2	A549	JEG-3
Doxorubicin	–	0.469	1.472	0.499	0.847
**1**	OH	0.374	0.629	2.709	0.310
**2**	OSO_2_CH_3_	0.528	0.419	1.250	0.182
**3**	OSO_2_NH_2_	0.448	0.301	0.365	2.974
**4**	OSO_2_N(CH_3_)_2_	1.279	1.137	>5	2.936
**5**	OBn	0.690	0.764	0.298	0.397

^a)^ IC_50_ for 48 h drug exposure at pH 7.4. Stock solution were prepared in DMSO and diluted into culture medium to give 1% final DMSO concentrations

C5-Hydroxy-1*H*-indol-2-ylcarbonyl-*seco*-CI (**1**, IC_50_: 0.374 µM) showed similar activity as doxorubicin (IC_50_: 0.374 µM) against the COLO 205 cell line and was more active than doxorubicin against both the SK-MEL-2 and A-549 cancer cell lines (IC_50_: 0.374 µM and 0.629 µM *vs.* 1.472 µM and 0.847 µM). Indole C5-methylsulfonyloxy derivative **2** showed more activity than doxorubicin against both the SK-MEL-2 and JEG-3 cancer cell lines (IC_50_: 0.419 µM and 0.182 µM). Indole C5-aminosulfonyloxy derivative **3** showed more activity than doxorubicin against the SK-MEL-2 cancer cell line (IC_50_: 0.301 µM). Overall, compounds **2** and **3** exhibited more activity than the others against the SK-MEL-2 cancer cell line. Indole C5-*N,N*-dimethylaminosulfonyloxy derivative **4** was less active than doxorubicin against all four cancer cell lines. As a result, it is thought that the indole C5-*N,N*-dimethylaminosulfonylamino substituent of *seco*-CI did not contribute to the embedded minor groove interaction. Indole C5-benzyloxy-1*H*-indole *seco*-CI **5** exhibited weaker activity than doxorubicin against the SK-MEL-2, A549 and JEG-3 cancer cell lines (IC_50_: 0.764 µM, 0.298 µM and 0.397 µM). Parrish *et al*. have reported that (+)-CBI-5-OH (1,2,9,9a-tetrahydrocyclopropa[*c*]benz[*e*]indol-4-one:CBI) and (+)-CBI-5-OBn were very active against the L1210 cancer cell line (IC_50_: 200 and 50 pM) [20]. This trend has been explained by the fact that inclusion of an extended aromatic unit in the CI unit, as in the case of CBI, significantly enhances chemical stability and cytotoxicity [21]. In conclusion, both the alkylation part (CBI unit) and a (+)-enantiomer are seem to be important factors for enhanced activity compared with *racemic* and a *seco-*CI unit. 

## 3. Experimental

### 3.1. General

Moisture- or air-sensitive reactions were conducted under nitrogen in distilled solvents. The commercial reagents were purchased from Aldrich, Fluka, or Sigma. Melting points were measured on a Gallenkamp melting point apparatus and are not corrected. ^1^H- (400 MHz), ^13^C-NMR (100 MHz), HSQC, and HMQC spectra were taken on a Varian AS 400 MHz spectrometer. Chemical shifts (δ) are in parts per million (ppm) relative to tetramethylsilane, and coupling constants (*J*) are in Hertz. GC/MS spectra were obtained on a Shimadzu MS-QP2010 mass spectrometer. The MPLC apparatus used was a Yamazen YFLC-AI-580. Analytical TLC was performed on pre-coated silica gel 60 F_254_ plates (Merck). Column chromatography was carried out on Merck silica gel 9385 (230-400 mesh) and the eluting solvent is indicated in each entry.

*Methyl 5-hydroxy-1H-indole-2-carboxylate* (**9**). According to the method of Fukuda *et al*. [3], to a solution of metal Na (2.2 g, 94.35 mmol) in methanol (100 mL) was added a solution of 3-hydroxy-benzaldehyde (**6**, 5.0 g, 23.58 mmol) and methyl azidoacetate (**7**, 10.9 g, 94.35 mmol) in methanol (100 mL) at −20 °C for 30 min. The reaction mixture was stirred at −20 °C for 3 h and at 0 °C for 12 h and then added with water to form a precipitate. This precipitate was filtered off and washed with ice water to obtain compound **8**, which was refluxed in xylene (50 mL) for 1 h. The remaining xylene was evaporated to yield the crude compound which was recrystallized from ethyl acetate/*n*-hexane to afford **9** as a pale pink powder. Yield*:* 3.3 g (42%); mp: 137–139 °C; ^1^H-NMR (CDCl_3_) δ*:* 3.94 (3H, s, OCH_3_), 4.78 (1H, s, OH), 6.94 (1H, dd, *J* = 8.8, 2.4 Hz, H-6), 7.07 (1H, d, *J* = 2.0 Hz, H-4), 7.09-7.10 (1H, m, H-3), 7.29 (1H, d, *J* = 8.8 Hz, H-7), 8.81 (1H, s, NH); ^13^C-NMR (CDCl_3_) δ*:* 52.0 (OCH_3_), 105.9 (C-4), 108.0 (C-3), 112.7 (C-7), 116.2 (C-6), 127.9 (C-7a), 128.1 (C-2), 132.3 (C-3a), 150.1 (C-5), 162.4 (C=O); GC-MS (EI) *m/z:* 191 [M]^+^ , 176 [M-CH_3_] ^+^.

*5-Hydroxy-1H-indole-2-carboxylic acid* (**10**). A suspension of methyl 5-hydroxy-1*H*-indole-2-carboxylate (**9**, 1 g, 5.23 mmol) in methanol (10 mL) was treated with a solution of 10 % NaOH (10 mL) and heated at 60 °C for 1 h. After cooling to room temperature, the solution was acidified with 10% HCl and the precipitate formed was isolated by filtration and washed with water to give the crude compound which was recrystallized with methanol/ethyl acetate to afford **10** as a white powder. Yield: 908 mg (98%); mp: 245–247 (dec.) °C, (249 °C (dec.), commercial available compound). 

*3-Benzyloxybenzaldehyde* (**11**). According to the method of Schmidhammer *et al*. [26], a suspension of K_2_CO_3_ (14.8 g, 107.13 mmol) in chloroform/methanol (2:1, 300 mL) was refluxed for 15 min and added a mixture of **6** (3.0 g, 24.57 mmol) and benzyl bromide (5.0 g, 29.48 mmol) in chloroform/ methanol (2:1, 90 mL) over 30 min. The reaction mixture was refluxed for 8 h to yield a precipitate which was filtered and the residue was concentrated to give a crude oily compound. This was diluted with dichloromethane and the organic solvent was washed with brine, dried with anhydrous MgSO_4_, filtered and concentrated to give the crude compound which was recrystallized from ethyl acetate/*n*-hexane to give **11** as a white powder. Yield: 5.2 g (99%); mp: 54–56 °C (56–58 °C) [27].

*Methyl 7-benzyloxy-1H-indole-2-carboxylate* (**13**) and *methyl 5-benzyloxy-1H-indole-2-carboxylate* (**14**). To a solution of Na (2.2 g, 94.35 mmol) in absolute methanol (100 mL) was added a solution of 3-benzyloxybenzaldehyde (**7**, 5.0 g, 23.58 mmol) and methyl azidoacetate (**7**, 10.9 g, 94.35 mmol) in methanol (100 mL) at −20 °C, and the mixture was stirred at 0 °C for 12 h. After addition of cold water, the resulting precipitate was collected by filtration. The solid was washed with water and dried to give methyl 2-azido-3-(3-benzyloxyphenyl)acrylate (**12**, 6.4 g, 88 %) as pale yellow crystals. To refluxing xylene (50 mL) was added a xylene solution (50 mL) of acrylate (6.4 g) and the mixture was refluxed for 1 h. After cooling, the solvent was removed *in vacuo* to give the crude oily compound which was purified by column chromatography (ethyl acetate/*n*-hexane = 1:9) to afford methyl 7-benzyloxy-1*H*-indole-2-carboxylate (**13**) and methyl 5-benzyloxy-1*H*-indole-2-carboxylate (**14**) as white crystals. Compound **13**: Yield: 1.6 g (40%); mp: 130–131 °C (132–133 °C) [28]; Compound **14**: Yield: 1.8 g (45%); mp: 156–157 °C; ^1^H-NMR (CDCl_3_) δ: 3.94 (3H, s, OCH_3_), 5.10 (2H, s, OCH_2_), 7.03 (1H, dd, *J* = 8.8, 2.4 Hz, H-6), 7.13 (1H, t, *J* = 2.0 Hz, H-3), 7.15 (1H, d, *J* = 2.4 Hz, H-4), 7.31-7.47 (6H, m, H-7 and Ar-H×5), 8.99 (1H, s, NH); ^13^C-NMR (CDCl_3_) δ: 52.2 (OCH_3_), 70.9 (OCH_2_), 104.4 (C-6), 108.6 (C-3), 113.0 (C-4), 117.9 (C-7), 128.0 (C-2), 132.6 (C-7a), 127.8 (Ar-C), 128.1 (Ar-C), 128.8 (Ar-C), 129.0 (C-3a), 137.5 (Ar-C), 154.1 (C-5), 162.6 (CO); GC-MS (EI) *m/z*: 281 [M]^+^, 190 [M-CH_2_C_6_H_5_]^+^.

*5-Benzyloxy-1H-indole-2-carboxylic acid (***15***)*. A suspension of methyl 5-benzyloxy-1*H*-indole-2-carboxylate (**14**, 1 g, 3.56 mmol) in methanol (10 mL) was reacted with a solution of 10 % NaOH (10 mL) and heated at 60 °C for 1 h. The resulting mixture was worked up conditions like compound **10** to afford **15** as a white powder. Yield: 930 mg (98%); mp: 193–195 °C (192 °C, commercially available compound) 

*5-Benzyloxy-1-chloromethyl-1,2-dihydro-3-[(5-hydroxy-1H-indol-2-yl)carbonyl]indoline* (**17**). 5-Benzyloxy-1-chloromethyl-*N*-(*tert*-butyloxycarbonyl)indoline (**16**, 50 mg, 0.13 mmol) [22] was dissolved in 4M HCl in ethyl acetate (5 mL) and stirred at room temperature for 1 h. The reaction mixture was treated with 20% NaOH and extracted with ethyl acetate and the organic layer was dried with anhydrous MgSO_4_ and filtered, concentrated to give the residue which was dried under vacuum for 1 h. The resulting amine hydrochloride (5-benzyloxy-1-chloromethyl-3*H*-indoline·HCl) was dissolved in dimethylformamide (2 mL) and 5-substituted-1*H*-indole-2-carboxylic acid (50.4 mg, 0.20 mmol) and EDCI (74.0 mg, 0.40 mmol) were added. The reaction mixture was stirred for 14 h at room temperature and added with water (3 mL) and obtained precipitates were purified by column chromatography (ethyl acetate:*n*-hexane=1:5) to afford **17** as a white solid. Yield*:* 39 mg (67%); mp: 194–196 °C; ^1^H NMR (400 MHz, CD_3_Cl) δ: 3.55–3.60 (1H, m, CH_2_Cl), 3.76–3.82 (2H, m, CH_2_Cl, H-1), 4.47 (1H, dd, *J* = 10.6, 4.0 Hz, H-2a), 4.63–4.68 (1H, m, H-2b), 5.09 (2H, s, OCH_2_), 6.72 (1H, dd, *J* = 8.4, 2.4 Hz, H-6), 6.89 (1H, d, *J* = 2.0 Hz, H-3'), 6.94 (1H, dd, *J* = 8.8, 2.4 Hz, H-6'), 7.09 (1H, d, *J* = 2.0 Hz, H-4'), 7.17 (1H, d, *J* = 8.0 Hz, H-7), 7.30-7.45 (6H, m, Ar-H×5, H-7), 8.05 (1H, s, H-4), 8.31 (1H, s, OH), 10.03 (1H, s, NH); ^13^C NMR (100 MHz, CD_3_Cl) δ*:* 42.9 (C-1), 47.2 (CH_2_Cl), 54.9 (C-2), 70.2 (OCH_2_), 104.8 (C-4), 105.2 (C-4'), 105.4 (C-3'), 111.1 (C-6), 112.6 (C-7'), 116.4 (C-6'), 123.6 (C-7a), 124.6 (C-7) 127.5 (Ar-C), 127.9 (Ar-C), 128.5 (Ar-C), 130.5 (C-2'), 131.2 (C-7a'), 136.8 (Ar-C), 144.9 (C-3a), 151.5 (C-5'), 159.4 (C-5), 160.7 (CO); GC-MS (EI) *m/z:* 432 [M]^+^, 434 [M+2] ^+^.

*Chloromethyl-5-hydroxy-1,2-dihydro-3-[(5-hydroxy-1H-indol-2-yl)carbonyl]indoline* (**1**). 5-Benzyloxy-1-chloromethyl-1,2-dihydro-3-[(5-hydroxy-1*H*-indol-2-yl)carbonyl]indoline (**17**, 20 mg, 46.30 μmol) was added a suspension of 10% Pd/C (125 mg) in acetone (10 mL) to a Parr bottle. After catalytic hydrogenation at 55 psi for 1 h, the reaction mixture was filtered and the filtrate was evaporated to yield **1** as a white solid. Yield: 12 mg (76%); mp*:* 175–176 °C; ^1^H-NMR (CD_3_OD) δ*:* 3.54 (1H, dd, *J =* 11.0, 8.2 Hz, CH_2_Cl), 3.64–3.68 (1H, m H-1), 3.76 (1H, dd, *J =* 10.8, 4.4 Hz, CH_2_Cl), 4.33 (1H, dd, *J =* 11.0, 4.6 Hz, H-2a), 4.54 (1H, dd, *J =* 10.8, 9.2 Hz, H-2b), 6.45 (1H, dd, *J =* 8.4, 2.4 Hz, H-6), 6.75 (1H, dd, *J =* 8.8, 2.4 Hz, H-6'), 6.86 (1H, s, H-3'), 6.92 (1H, d, *J =* 2.4 Hz, H-4') 7.07 (1H, d, *J =* 8.0 Hz, H-7), 7.20 (1H, d, *J =* 8.8 Hz, H-7'), 7.56 (1H, s, H-4); ^13^C-NMR (CD_3_OD) δ: 42.8 (C-1), 47.7 (CH_2_Cl), 55.2 (C-2), 104.8 (C-4), 105.3 (C-4'), 105.4 (C-3'), 111.2 (C-6), 112.4 (C-7'), 115.6 (C-6'), 123.1 (C-7a), 124.8 (C-7), 128.6 (C-3a'), 130.8 (C-2'), 131.9 (C-7a'), 144.7 (C-3a), 151.2 (C-5'), 157.8 (C-5), 161.5 (CO); GC-MS (EI) *m/z:* 342 [M]^+^, 344 [M+2] ^+^.

*5-Benzyloxy-1-chloromethyl-1,2-dihydro-3-[(5-methylsulfonyloxy-1H-indol-2-yl)carbonyl]indoline* (**18**). To the solution of **17** (50 mg, 0.12 mmol) in CH_2_Cl_2_ (5 mL) was added methanesulfonyl chloride (26.4 mg, 0.23 mmol) and triethylamine (35.4 mg, 0.35 mmol) at room temperature. The reaction mixture was stirred for 4 h, water was added, followed by extraction CH_2_Cl_2_ and drying with anhydrous MgSO_4_. After concentration, the residue was purified by column chromatography (ethyl acetate/*n*-hexane = 1:5) to yield **18** as a white solid. Yield*:* 48 mg (92%); mp*:* 203–206 °C; ^1^H-NMR (CD_3_COCD_3_) δ*:* 3.27 (3H, s, CH_3_), 3.82–4.02 (3H, m, CH_2_Cl, H-1), 4.55 (1H, dd, *J =* 10.8, 4.4 Hz, H-2a), 4.82 (1H, t, *J =* 9.8 Hz, H-2b), 5.15 (2H, s, OCH_2_), 6.79 (1H, d, *J =* 8.4 Hz, H-6), 7.28-7.52 (9H, m, Ar-H×5, H-3', 6, 6', 7), 7.67 (1H, d, *J =* 8.4 Hz, H-7'), 7.72 (1H, s, H-4'), 8.05 (1H, s, H-4), 11.11 (1H, s, NH); ^13^C-NMR (CD_3_COCD_3_) δ*:* 32.0 (CH_3_), 42.7 (C-1), 47.2 (CH_2_Cl), 50.3 (C-2), 70.0 (OCH_2_), 105.2 (C-4), 106.1 (C-4'), 106.2 (C-3'), 110.6 (C-6), 112.7 (C-7'), 115.2 (C-6'), 119.5 (C-7a), 124.6 (C-7) 127.8 (Ar-C), 128.0 (C-3a'), 128.2 (Ar-C), 128.6 (Ar-C), 130.9 (C-2'), 135.8 (C-7a'), 137.6 (Ar-C), 144.0 (C-3a), 145.4 (C-5'), 159.5 (C-5), 160.1 (CO); GC-MS (EI) *m/z:* 510 [M]^+^, 420 [M- CH_2_C_6_H_5_]^+^.

*5-Benzyloxy-1-chloromethyl-1,2-dihydro-3-[(5-sulfamoyloxy-1H-indol-2-yl)carbonylindoline* (**19**). According to method by Okada *et al*. [29], formic acid (8.5 mg, 0.18 mmol) and chlorosulfonyl isocyanate (26.2 mg, 0.18 mmol) were allowed to react with each other at 80 °C till evolution of carbon dioxide gas ceased to thus form aminosulfonyl chloride. To a solution of **17** (40 mg, 0.09 mmol) in dimethylacetamide (DMA, 3 mL) was added the aminosulfonyl chloride and the resulting mixture left to stir for 3 h. The solution was treated with water, extracted with CH_2_Cl_2_ and the organic layer was dried with anhydrous MgSO_4_. After concentration, the residue was purified by column chromatography (ethyl acetate/*n*-hexane=1:5) to yield **19** as a white solid. Yield*:* 30 mg (62%); mp*:* 196–197 °C; ^1^H-NMR (CD_3_OD) δ*:* 3.60 (1H, dd, *J =* 10.6, 7.4 Hz, CH_2_Cl), 3.70 (1H, m, H-1), 3.78 (1H, dd, *J =* 10.6, 4.2 Hz, CH_2_Cl), 4.32 (1H, dd, *J =* 11.0, 4.6 Hz, H-2a), 4.55 (1H, dd, *J =* 10.8, 9.2 Hz, H-2b), 4.96 (2H, s, OCH_2_), 6.64 (1H, dd, *J =* 8.8, 2.4 Hz, H-6), 6.76 (1H, dd, *J =* 8.8, 2.4 Hz, H-6'), 6.86 (1H, s, H-3'), 6.92 (1H, d, *J =* 2.4 Hz, H-4'), 7.16 (1H, d, *J =* 8.4 Hz, H-7), 7.20–7.34 (6H, m, Ar-H×5, H-7'), 7.78 (1H, s, H-4); ^13^C-NMR (CD_3_OD) δ*:* 42.6 (C-1), 47.3 (CH_2_Cl), 55.1 (C-2), 70.1 (OCH_2_), 104.9 (C-4), 105.0 (C-4'), 105.4 (C-3'), 110.8 (C-6), 112.7 (C-7'), 115.8 (C-6'), 124.7 (C-7a), 125.0 (C-7) 127.6 (Ar-C), 127.9 (Ar-C), 128.5 (Ar-C), 128.7 (C-3a'), 130.9 (C-2'), 131.8 (C-7a'), 137.5 (Ar-C), 145.0 (C-3a), 151.4 (C-5'), 159.4 (C-5), 161.4 (CO); GC-MS (EI) *m/z:* 432 [M-SO_2_NH_2_] ^+^, 434 [M-SO_2_NH_2_+2] ^+^.

*5-Benzyloxy-1-chloromethyl-1,2-dihydro-3-[(5-dimethylsulfamoyloxy-1H-indol-2-yl)carbonyl]indoline* (**20**). To the solution of **17** (40 mg, 0.09 mmol) in CH_2_Cl_2_ (5 mL) was added dimethylsulfamoyl chloride (26.56 mg, 0.18 mmol) and triethylamine (35.4 mg, 0.35 mmol) at room temperature, and the mixture was stirred for 18 h. The solution was treated with water, extracted with CH_2_Cl_2_ and the organic layer was dried with anhydrous MgSO_4_. After concentration, the residue was purified by column chromatography (ethyl acetate/*n*-hexane=1:5) to yield **20** as a pale yellow solid. Yield*:* 36 mg (72%); mp*:* 116–117 °C; ^1^H-NMR (CD_3_COCD_3_) δ*:* 2.97 (6H, s, CH_3_×2), 3.82–4.01 (3H, m, CH_2_Cl, H-1), 4.54 (1H, dd, *J =* 10.8, 3.6 Hz, H-2a), 4.81 (1H, t, *J =* 10.0 Hz, H-2b), 5.14 (2H, s, OCH_2_), 6.79 (1H, d, *J =* 9.2 Hz, H-6), 7.26-7.51 (8H, m, Ar-H×5, H-3', 6', 7), 7.65 (1H, d, *J =* 8.8 Hz, H-7'), 7.69 (1H, s, H-4'), 8.05 (1H, s, H-4), 11.09 (1H, s, NH); ^13^C-NMR (CD_3_COCD_3_) δ*:* 38.3 (CH_3_×2), 42.8 (C-1), 47.7 (CH_2_Cl), 54.8 (C-2), 70.0 (OCH_2_), 105.3 (C-4), 106.2 (C-4'), 106.3 (C-3'), 110.5 (C-6), 113.3 (C-7'), 114.9 (C-6'), 124.6 (C-7a), 125.1 (C-7) 127.8 (Ar-C), 127.9 (Ar-C), 128.2 (C-3a'), 128.6 (Ar-C), 132.9 (C-2'), 134.8 (C-7a'), 137.7 (Ar-C), 144.6 (C-3a), 145.4 (C-5'), 159.5 (C-5), 160.1 (CO); GC-MS (EI) *m/z:* 540 [M]^+^, 542 [M+2] ^+^.

*1-Chloromethyl-5-hydroxy-1,2-dihydro-3-[(5-methylsulfonyloxy-1H-indol-2-yl)carbonyl]indoline* (**2**). To solution of 10% Pd-C (10 mg) in acetone (10 mL) was added **18** (20 mg, 39.21 μmol) and the resulting mixture was worked up conditions like compound **1** to give compound **2** as a white solid. Yield*:* 8 mg (50%); mp*:* 155–158 °C; ^1^H-NMR (CD_3_COCD_3_) δ*:* 2.80 (3H, s, CH_3_), 3.77–3.98 (3H, m, CH_2_Cl, H-1), 4.49 (1H, dd, *J =* 10.2, 4.2 Hz, H-2a), 4.74 (1H, t, *J =* 9.6 Hz, H-2b), 6.58 (1H, d, *J =* 9.2 Hz, H-6), 6.90 (1H, d, *J =* 8.0 Hz, H-6'), 7.01 (1H, s, H-3’), 7.08 (1H, s, H-4’), 7.24 (1H, d, *J =* 8.4 Hz, H-7), 7.42 (1H, d, *J =* 9.2 Hz, H-7’), 7.87 (1H, s, H-4), 8.34 (1H, s, OH), 10.59 (1H, s, NH); ^13^C-NMR (CD_3_COCD_3_) δ*:* 36.5 (CH_3_), 42.8 (C-1), 47.8 (CH_2_Cl), 54.9 (C-2), 105.6 (C-4), 106.0 (C-4'), 106.1 (C-3'), 111.2 (C-6), 113.5 (C-7'), 115.1 (C-6'), 122.8 (C-7a), 125.1 (C-7), 128.1 (C-3a'), 133.0 (C-2'), 134.9 (C-7a'), 144.0 (C-3a), 145.2 (C-5'), 158.1 (C-5), 160.0 (CO); GC-MS (EI) *m/z:* 420 [M]^+^, 342 [M-SO_2_CH_3_]^+^. 

*1-Chloromethyl-5-hydroxy-1,2-dihydro-3-[(5-sulfamoyloxy-1H-indol-2-yl) carbonyl]indoline* (**3**). To a solution of 10% Pd-C (10 mg) in acetone (10 mL) was added **20** (20 mg, 39.06 μmol) and the resulting mixture was worked up conditions like compound **1**. Compound **3** was obtained as a white solid. Yield*:* 8.8 mg (53%); mp*:* 195–197 °C; ^1^H-NMR (CD_3_COCD_3_) δ*:* 3.77–3.98 (3H, m, CH_2_Cl, H-1), 4.48 (1H, d, *J =* 10.8 Hz, H-2a), 4.74 (1H, t, *J =* 9.6 Hz, H-2b), 6.69 (1H, d, *J =* 8.4 Hz, H-6), 6.91 (1H, d, *J =* 8.8 Hz, H-6'), 7.02 (1H, s, H-3'), 7.09 (1H, s, H-4'), 7.24 (1H, d, *J =* 8.0 Hz, H-7), 7.42 (1H, d, *J =* 8.4 Hz, H-7'), 7.88 (1H, s, H-4), 8.38 (1H, s, OH), 10.61 (1H, s, NH); ^13^C-NMR (CD_3_COCD_3_) δ*:* 43.5 (C-1), 48.5 (CH_2_Cl), 55.5 (C-2), 105.8 (C-3', C-4'), 106.3, (C-4), 111.6 (C-6), 113.6 (C-7'), 116.5 (C-6'), 123.6 (C-7a), 125.7 (C-7), 129.7 (C-3a'), 132.1 (C-2'), 132.3 (C-7a'), 146.2 (C-3a), 152.5 (C-5'), 158.7 (C-5), 161.1 (CO); GC-MS (EI) *m/z:* 342 [M-SO_2_NH_2_] ^+^, 344 [M+2-SO_2_NH_2_] ^+^.

*1-Chloromethyl-5-hydroxy-1,2-dihydro-3-[(5-dimethylsulfamoyloxy-1H-indol-2-yl)carbonyl]indoline* (**4**). To solution of 10% Pd-C (5 mg) in acetone (10 mL) was added **20** (20 mg, 37.03 μmol) and the resulting mixture was worked up conditions like compound **1**. Compound **4** was obtained as a white solid. Yield*:*11 mg (66%); mp*:* 217–218 °C; ^1^H NMR (CD_3_COCD_3_) δ*:* 2.94 (6H, s, CH_3_×2), 3.75–3.96 (3H, m, CH_2_Cl, H-1), 4.49 (1H, dd, *J =* 11.0, 4.2 Hz, H-2a), 4.75 (1H, t, *J =* 10.0 Hz, H-2b), 6.58 (1H, d, *J =* 7.6 Hz, H-6), 7.22 (3H, d, *J =* 7.2 Hz, H-3', 6', 7), 7.61 (1H, d, *J =* 8.8 Hz, H-7'), 7.66 (1H, s, H-4'), 7.85 (1H, s, H-4), 8.37 (1H, s, OH), 11.01 (1H, s, NH); ^13^C-NMR (CD_3_COCD_3_) δ*:* 38.2 (CH_3_×2), 42.8 (C-1), 47.8 (CH_2_Cl), 54.8 (C-2), 105.6 (C-4), 106.1 (C-4'), 106.2 (C-3'), 111.2 (C-6), 113.2 (C-7'), 114.9 (C-6'), 119.3 (C-7a), 123.0 (C-7), 125.1 (C-3a'), 133.0 (C-2'), 134.8 (C-7a'), 144.6 (C-3a), 145.3 (C-5'), 158.0 (C-5), 159.9 (CO); GC-MS (EI) *m/z:* 449 [M]^+^, 451 [M+2] ^+^.

*1-Chloromethyl-5-hydroxy-N-(tert-butyloxycarbonyl)-indoline (21)* To a solution of 10% Pd-C (25 mg) in methanol (10 mL) was added **16** (100 mg, 0.27 mmol) and the resulting mixture was worked up like compound **1**. Compound **21** was obtained as a white solid. Yield*:* 74 mg (98%); mp*:* 178–179 °C; ^1^H- NMR (CDCl_3_) δ*:* 1.56 (9H, s, C(CH_3_)_3_), 3.47 (1H, t, *J* = 9.8 Hz, CH_2_Cl), 3.54–3.61 (1H, m, H-1), 3.69 (1H, dd, *J* = 10.4, 3.6 Hz, CH_2_Cl), 3.90–3.93 (1H, m, H-2), 4.07–4.12 (1H, m, H-2), 6.48 (1H, dd, *J* = 8.4, 2.0 Hz, H-6), 7.01 (1H, d, *J* = 8.0 Hz, H-7), 7.47 (1H, s, H-4); ^13^C-NMR (CDCl_3_) δ*:* 28.4 (C(CH_3_)_3_), 41.8 (C-1), 47.5 (CH_2_Cl), 53.0 (C-2), 81.5 (C(CH_3_)_3_), 103.0 (C-4), 109.6 (C-6), 122.1 (C-7a), 125.0 (C-7), 143.8 (C-3a), 152.6 (C-5), 157.0 (CO); GC-MS (EI) *m/z:* 183 [M-BOC] ^+^, 185 [M+2-BOC] ^+^.

*1-Chloromethyl-5-hydroxy-1,2-dihydro-3-[(5-benzyloxy-1H-indol-2-yl)carbonyl]indoline* (**5**). Compound **21** (50 mg, 0.18 mmol) was dissolved in 4M HCl in ethyl acetate (5 mL) and stirred at room temperature for 1 h. The reaction mixture was treated with 20% NaOH and extracted with ethyl acetate and the organic layer was dried with anhydrous MgSO_4_, filtered and concentrated to give a residue which was dried under vacuum for 1 h. The resulting amine hydrochloride (5-hydroxy-1-chloromethyl-3*H*-indoline·HCl) was dissolved in dimethylformamide (2 mL) and 5-benzyloxy-1*H*-indole-2-carboxylic acid (70.6 mg, 0.26 mmol) and EDCI (101.0 mg, 0.5 mmol) were added. The reaction mixture was stirred for 14 h at room temperature and then treated with water (3 mL) to form a precipitate. This precipitate was purified by column chromatography (ethyl acetate/*n*-hexane = 1:5) to afford **5** as a white solid. Yield*:* 63.1 mg (83%); mp*:* 220–222 °C; ^1^H-NMR (CD_3_COCD_3_) δ*:* 3.78 (1H, t, *J* = 8.0 Hz, CH_2_Cl), 3.86 (1H, m, H-1), 3.96 (1H, dd, *J* = 10.4, 3.6 Hz, CH_2_Cl), 4.49 (1H, dd, *J* = 10.8, 4.4 Hz, H-2a), 4.74 (1H, t, *J* = 10.0 Hz, H-2b), 5.15 (2H, s, OCH_2_), 6.59 (1H, d, *J* = 8.0 Hz, H-6), 7.04 (1H, d, *J* = 8.4 Hz, H-6'), 7.09 (1H, s, H-3'), 7.23–7.52 (8H, m, Ar-H×5, H-4', H-7, H-7'), 7.88 (1H, s, H-4), 8.38 (1H, s, OH), 10.74 (1H, s, NH); ^13^C-NMR (CD_3_COCD_3_) δ*:* 43.5 (C-1), 48.5 (CH_2_Cl), 55.5 (C-2), 71.0 (OCH_2_), 104.8 (C-4), 106.2 (C-4'), 106.3 (C-3'), 111.6 (C-6), 113.9 (C-7'), 117.3 (C-6'), 123.6 (C-7a), 125.7 (C-7) 127.5 (Ar-C), 127.9 (Ar-C), 128.5 (Ar-C), 132.2 (C-2'), 132.8 (C-7a'), 138.9 (Ar-C), 146.1 (C-3a), 154.5 (C-5'), 158.6 (C-5), 161.0 (CO); GC-MS (EI) *m/z:* 432 [M]^+^, 434 [M+2] ^+^.

### 3.2. Cytotoxicity

Synthetic compounds were dissolved in DMSO to obtain 10 mM stock solutions, which were diluted with RPMI 1640 to obtain 100 µM solutions. These solutions were then further diluted with 1% DMSO in RPMI 1640 to prepare solutions from concentrations of 50 µM to 10 nM. These compound solutions (10 µL) were added to wells containing 90 µL media/cell suspension.

The human cancer cells (COLO 205, SK-MEL-2, A549, and JEG-3) were purchased from the Korean cell line bank (KCLB). These cancer cell lines were grown in RPMI 1640 [(+) L-glutamine, (+) 25 mM HEPES, Gibco], except JEG-3 cells, which were grown in Delbecco’s Modified Eagle Medium (DMEM) supplemented with 10% fetal bovine serum (Gibco) and 1% penicillin/streptomycin (Gibco). Cells were maintained at 37 °C in a 5% humidified CO_2_ atmosphere. 

Cell concentrations were adjusted to 5 × 10^4^ cells/mL (COLO 205, SK-MEL-2), 1 × 10^5^ cells/mL (A549) and 2 × 10^4^ cells/mL (JEG-3) after counting the cells with a hemocytometer. Cell suspentions (90 µL) were seeded onto 96-well flat-bottom cell culture plates. After 24 h in a 5% CO_2_ atmosphere at 37 °C, the drug solutions diluted 1% DMSO in RPMI 1640 were added to each well (10 µL/well). The plates were incubated for 48 h at 37 °C in a 5% CO_2_ atmosphere. MTT (3-(4,5-dimethylthiazol-2-yl)-2,5-diphenyl tetrazolium bromide) was dissolved in PBS (2 mg/mL). After the indicated cell incubation period, 20 μL of this stock MTT solution was added to each well and the plates were further incubated for 4 h at 37 °C in a 5% CO_2_. The contents of the well were shaken and the plates were read on an ELISA reader, utilizing Sunrise xfluor4 V.4.51, with a test wavelength of 570 nm. The dose inhibiting the growth by 50% (IC_50_) was extrapolated from curves generated based of the average of the absorbance data (seven points/concentration).

## 4. Conclusions

Indole C5-*O*-substitued *seco*-CI derivatives **1**-**5** were synthesized and evaluated against four cancer cell lines. From the *in vitro* cytotoxic test data, compounds **1**-**5** showed more potency than doxorubicin against the SK-MEL-2 cell line and compounds **1**, **2** and **5** more weakly potent than doxorubicin against the JEG-3 cancer cell line, and the indole C5-*N,N*-dimethylaminosulfonyl derivative **4** was inactive against all cancer cell lines. It is thought that the indole C5-*N,N*-dimethylaminosulfonylamino substituent of *seco*-CI did not contribute to the embedded minor groove interaction.
